# A Systematic Review of the Accuracy, Validity, and Reliability of Markerless Versus Marker Camera-Based 3D Motion Capture for Industrial Ergonomic Risk Analysis

**DOI:** 10.3390/s25175513

**Published:** 2025-09-04

**Authors:** Sofia Scataglini, Eugenia Fontinovo, Nouran Khafaga, Muhammad Ubaidullah Khan, Muhammad Faizan Khan, Steven Truijen

**Affiliations:** 1 4D4ALL Lab, Department of Rehabilitation Sciences and Physiotherapy, Center for Health and Technology (CHaT), Faculty of Medicine and Health Sciences, MOVANT, University of Antwerp, 2000 Antwerpen, Belgium; s1102455@studenti.univpm.it (E.F.); nouran.khafaga@student.uantwerpen.be (N.K.); muhammad.khan3@student.uantwerpen.be (M.U.K.); muhammadfaizan.khan@student.uantwerpen.be (M.F.K.); 2 Department of Industrial Engineering and Mathematical Sciences, Università Politecnica delle Marche, via Brecce Bianche 12, 60131 Ancona, Italy

**Keywords:** markerless 3D camera-based motion capture, marker-based 3D motion capture, ergonomic risk assessment, industrial workers, accuracy, reliability, validity

## Abstract

**Highlights:**

**What are the main findings?**

- Markerless camera-based motion capture systems (MCBSs) for ergonomic risk assessment in industrial settings offer substantial accuracy and reliability.
- Markerless systems (MCBSs) provide a feasible, scalable alternative to traditional ergonomic methods.

**What is the implication of the main finding?**

- Markerless systems (MCBSs) have strong potential for practical, real-world applications and automation.
- Markerless systems (MCBS) support Industry 5.0 goals in occupational risk prevention.

**Abstract:**

Ergonomic risk assessment is crucial for preventing work-related musculoskeletal disorders (WMSDs), which often arise from repetitive tasks, prolonged sitting, and load handling, leading to absenteeism and increased healthcare costs. Biomechanical risk assessment, such as RULA/REBA, is increasingly being enhanced by camera-based motion capture systems, either marker-based (MBSs) or markerless systems (MCBSs). This systematic review compared MBSs and MCBSs regarding accuracy, validity, and reliability for industrial ergonomic risk analysis. A comprehensive search of PubMed, WoS, ScienceDirect, IEEE Xplore, and PEDro (31 May 2025) identified 898 records; after screening with PICO-based eligibility criteria, 20 quantitative studies were included. Methodological quality was assessed with the COSMIN Risk of Bias tool, synthesized using PRISMA 2020, and graded with EBRO criteria. MBSs showed the highest precision (0.5–1.5 mm error) and reliability (ICC > 0.90) but were limited by cost and laboratory constraints. MCBSs demonstrated moderate-to-high accuracy (5–20 mm error; mean joint-angle error: 2.31° ± 4.00°) and good reliability (ICC > 0.80), with greater practicality in field settings. Several studies reported strong validity for RULA/REBA prediction (accuracy up to 89%, κ = 0.71). In conclusion, MCBSs provide a feasible, scalable alternative to traditional ergonomic assessment, combining reliability with usability and supporting integration into occupational risk prevention.

## 1. Introduction

Work-related musculoskeletal disorders (WMSDs) are one of the main problems affecting workers in industrial scenarios [1,2]. These are often caused by prolonged sitting, repetitive tasks, handling loads, and the use of vibrating tools [3]. These factors contribute substantially to absenteeism, reduced productivity, and increased healthcare costs. Industry 5.0 is adapting risk analysis tools to prevent WMSD risks [4] using observational methods such as Rapid Upper Limb Assessment (RULA) [5] and Rapid Entire Body Assessment (REBA) [6] with or without camera-based technologies, including marker-based (MBSs) and markerless systems (MCBSs) [7], to score WMSD risks. Camera-based tools can help to quantify WMSD risks using information that comes from the kinematics of workers during specific tasks (such as lifting, pulling, etc.) and be combined with traditional observational methods (e.g., RULA and REBA). However, MBSs and MCBSs are different. Marker-based motion capture systems (MBSs) are considered the gold-standard tools [8,9] in biomechanics, although the placement of markers on the body is time-consuming and sensitive to skin movement artifacts, limiting the natural free movement of workers. In contrast, markerless camera-based motion capture systems (MCBSs) do not require any markers, making their application less time-consuming and preserving natural movements and reducing skin movement artifacts [10]. Therefore, MCBSs can better reflect real-world industrial scenarios while preserving workers’ well-being and performance. Nevertheless, MCBs and MCBSs require the placement of camera-based systems in working scenarios and are less portable compared to wearable mocap inertial systems. However, they have the advantage of being able to reproduce 3D body shapes and even 4D representations (3D + time) for dynamic motion analysis [11]. Studies indicate that MCBSs have accuracy levels comparable to those of traditional methods like the European Assembly Worksheet (EAWS) [12,13]; REBA/RULA; and joint-angle comparisons [14,15], whereby an average deviation of 2.31 ± 4.00° in joint kinematics is observed [16]. In 2018, Mehrizi et al. [16] demonstrated that MCBSs can accurately estimate 3D joint kinematics during symmetrical lifting, though they also acknowledged the need for further validation studies when applying these systems to more complex tasks such as asymmetrical lifting in workplace environments. Comparisons of the Microsoft Kinect V2 (MCBS) and Vicon Bonita (MBS) systems demonstrate that while MBSs are more accurate, some movements (e.g., trunk inclination) are estimated well by markerless alternatives, showing divergences of 2.08° and 3.67°. However, when it comes to compound motions, for example, arm anteversion with external rotation and axial rotation, these levels of precision start to diminish [17]. Notwithstanding their limitations, MCBSs enhance efficiency by curtailing dependence on direct observation and improving posture evaluation. In ergonomic assessments, MCBSs have shown a high accuracy level for screening upper limb movements, providing excellent inter-rater reliability for shoulder motions (ICC ≥ 0.97) and strong agreement for thoracic rotation (ICC = 0.92) [18]. However, assessments of knee valgus have demonstrated moderate reliability (ICC = 0.59–0.82), raising uncertainties regarding the accuracy of lower limb evaluations [19]. The validity of MCBSs in gait analysis has also been assessed recently, producing good agreement in sagittal plane kinematics but moderate reliability in coronal and transverse planes (ICC = 0.520–0.608) [20]. Although MBS approaches stand out for their accuracy, MCBSs offer flexibility and non-intrusiveness and provide clinical and workplace improvements. They enable objective movement assessment, real-time feedback, and customized interventions [7]. Furthermore, integrating motion capture with virtual reality increases engagement in rehabilitation and ergonomic training and enables virtual workplace prototyping with quantitative ergonomic risk assessment [21]. Despite all these advancements, certain gaps still exist in terms of accuracy, validity, and reliability, and they are the objective of this systematic review.

## 2. Materials and Methods

This systematic review was conducted in accordance with the PRISMA 2020 statement [22]. A structured protocol based on the PICO framework (see Table 1) was used to define inclusion and exclusion criteria, ensuring transparency and methodological consistency. This systematic review is registered in PROSPERO with the following ID number: CRD420251038205 [23].

### 2.1. Data Sources and Search Strategy

A comprehensive literature search was conducted on 31 May 2025 across five databases: PubMed, Web of Science (WoS), ScienceDirect, IEEE Xplore, and the Physiotherapy Evidence Database (PEDro). The search strategy integrated Medical Subject Headings (MeSH) and keyword combinations relating to ergonomic assessment, motion capture technologies, and measurement outcomes (accuracy, validity, and reliability). Boolean operators (AND/OR) and database-specific filters (e.g., date range, English language, and exclusion of reviews) were applied. The full search strategies are provided in Table 1, Table 2, Table 3, Table 4 and Table 5.

### 2.2. Eligibility Criteria

Studies were included if they (1) involved working-age adults (18–65) performing occupational tasks; (2) used MBSs, MCBSs, or traditional ergonomic assessments; (3) evaluated at least one of the following outcomes: accuracy, validity, or reliability; and (4) used a quantitative study design. Studies were excluded if they were qualitative, were conducted in non-occupational settings, or lacked relevant outcome metrics. Table 6 outlines the detailed inclusion/exclusion criteria. 

### 2.3. Study Selection

Records were imported into EndNote [24] for duplicate removal, followed by two-phase screening in Rayyan [25]. Titles and abstracts were screened by two independent reviewers; disagreements were resolved through discussion or third-party arbitration. Full texts of potentially eligible articles were retrieved and reviewed against the inclusion criteria.

### 2.4. Data Extraction

Data were extracted using a standardized form, including author, year, system type (MBS/MCBS), ergonomic tool used, outcomes measured, and key findings. Accuracy metrics (e.g., RMSE and angle deviation), validity statistics (e.g., Cohen’s kappa (κ) and expert agreement), and reliability scores (e.g., ICCs) were recorded.

### 2.5. Synthesis and Grading of Evidence

Outcomes were synthesized narratively. No meta-analysis was performed due to heterogeneity in study designs and reporting metrics. The Consensus-Based Standards for the Selection of Health Measurement Instruments (COSMIN) [26,27] was used for methodological quality appraisal. Evidence strength was graded using the Evidence-Based Guideline Development (EBRO) framework [28], based on study quality, consistency, and applicability.

## 3. Results

### 3.1. Study Selection

**Figure 1:**
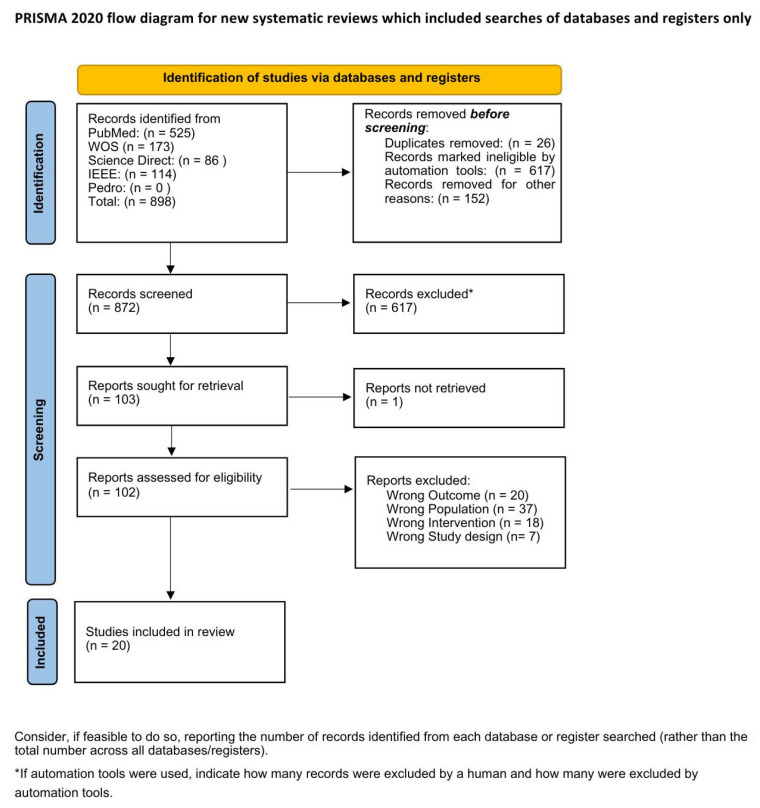
PRISMA 2020 flowchart.

A total of 898 records were identified from five databases: PubMed (525), ScienceDirect (86), Web of Science (173), IEEE Xplore (114), and PEDro (0). After removing 26 duplicates, 872 records remained. A total of 617 records were excluded automatically based on the initial screening, and 152 records were manually excluded. After reviewing 103 records for relevance, all 103 full-text articles were assessed for eligibility. Of these, 1 was not retrievable, and 82 studies were excluded for reasons including incorrect outcomes (20), publication type (7), population (37), and intervention (18). Finally, 20 studies [12,13,14,15,17,29,30,31,32,33,34,35,36,37,38,39,40,41,42,43] met the inclusion criteria and were included in the review, as shown in Figure 1.

### 3.2. Study Characteristics

The 20 studies included in the review were published between 2013 and 2025, with sample sizes ranging from 1 to 30 participants. Twelve studies included a direct comparison of MCBS technologies (e.g., Kinect, RealSense, and OpenPose) with MBS systems (e.g., Vicon, Optotrak, and BTS) [16,17,30,32,33,36,37,38,39,40,41,42]. One study evaluated a computer vision (CV)-based MCBS against a non-CV-based MCBS [31]. Five studies compared MCBSs with traditional ergonomic assessment methods (e.g., NIOSH, OCRA, REBA, EAWS, and RULA) [12,13,14,15,34]. The tasks analyzed ranged from lifting and handling to stationary workstation tasks in different workplace settings.

### 3.3. Evidence Table

The following table (Table 7) summarizes the study characteristics and key findings of the included studies, along with their measurement domains, such as accuracy, validity, and reliability. This table highlights the interventions used, the comparison methods, and the outcomes measured in each study, providing an overview of the research landscape.

### 3.4. Measurement Evidence Table

The following table (Table 8) summarizes the accuracy, validity, and reliability of the studies included in this review. It shows key metrics such as RMSE, Cohen’s kappa, and reliability statistics (ICCs) for the technologies assessed.

### 3.5. Risk of Bias in Included Studies

The methodological quality of the 20 included studies was assessed using the COSMIN Risk of Bias checklist, focusing on the evaluation of three measurement domains: reliability, validity, and accuracy. Assessments were performed independently by three reviewers. Discrepancies were resolved through consensus discussions.

Most studies demonstrated moderate to high methodological quality, particularly in the domains of accuracy and validity. However, reliability was the most inconsistently reported domain. Only six studies provided statistical reliability measures, such as intra-class correlation coefficients (ICCs), with values ranging from 0.70 to 0.99. Several studies referenced reliability qualitatively without formal assessment. Additionally, some studies were downgraded for small sample sizes, unclear statistical methods, or incomplete reporting of ergonomic scoring procedures.

Using COSMIN-based categorization:

- Very Good (V) quality was assigned to five studies [16,29,30,32,43], which employed robust statistical analyses and thorough validation.
- Adequate (A) quality applied to thirteen studies [13,14,15,31,33,34,36,37,38,39,40,41,42], generally due to minor limitations such as absence of inter-rater testing.
- Doubtful (D) or Inadequate (I) quality was noted in two studies [12,17], particularly those lacking sufficient methodological transparency or formal validation against reference tools.

A full summary of the risk of bias ratings by measurement of property and study is provided in Table 9.

### 3.6. Results of Individual Studies

Accuracy was reported in 18 studies, with MBSs consistently achieving joint-angle errors below 3°. MCBSs ranged in performance, with joint errors ranging from 4.4 to 23.4 cm (Liu et al. [33]) and RMSE values between 5° and 14° (Van Crombrugge et al. [40], Brunner et al. [17]). Validity was assessed in 19 studies (of which only 17 provided quantitative metrics) through agreement with expert ratings or MBSs. Agreement levels were high, with κ reaching up to 0.71 and the expert score alignment exceeding 70% in several cases. Only six studies addressed reliability statistically, with ICCs ranging from 0.70 to 0.99.

### 3.7. Summary of Synthesis and Bias

Synthesized findings showed that MBSs provided the highest accuracy and reliability but required controlled lab environments. MCBSs were more accessible and affordable, demonstrating acceptable accuracy and strong validity, particularly in complex dynamic tasks such as asymmetrical lifting and complex combined shoulder motions (e.g., anteversion with external rotation). Studies varied in sample size, ergonomic frameworks, and reporting detail. The risk of bias was lowest in studies that used standardized scoring systems and reference comparisons.

### 3.8. Reporting Bias

No specific publication bias was detected. However, there may be an overrepresentation of studies with positive findings due to underreporting of failed validation or low-accuracy results.

### 3.9. Certainty of Evidence

Based on COSMIN [27] and EBRO [28] assessments, the certainty of evidence for the accuracy and validity of outcomes was moderate to high. However, the certainty for reliability was lower due to inconsistent reporting and lack of statistical confirmation in most studies. Using the EBRO classification system [28], the level of evidence for each key measurement property—accuracy, validity, and reliability—was determined based on the number of studies, the methodological quality (assessed via COSMIN [26,27]), and the consistency of the findings across the studies. A total of 20 studies were included in this review, with 18 studies providing quantitative accuracy metrics. Two studies (Eldar, 2020 [15] and Otto et al. [12]) did not report numerical accuracy data and were therefore excluded from the EBRO grading for accuracy (Table 10).

Table 10 presents a summary of the EBRO levels of evidence for accuracy, validity, and reliability. It shows the number of studies evaluated for each measurement property, along with the corresponding EBRO levels and the associated certainty levels. Accuracy is rated A2 with a high certainty level, validity is rated B with a moderate certainty level, and reliability is rated C with a low certainty level. These levels reflect both the quantity and consistency of evidence available and should be considered when interpreting the results of this review.

**Figure 2:**
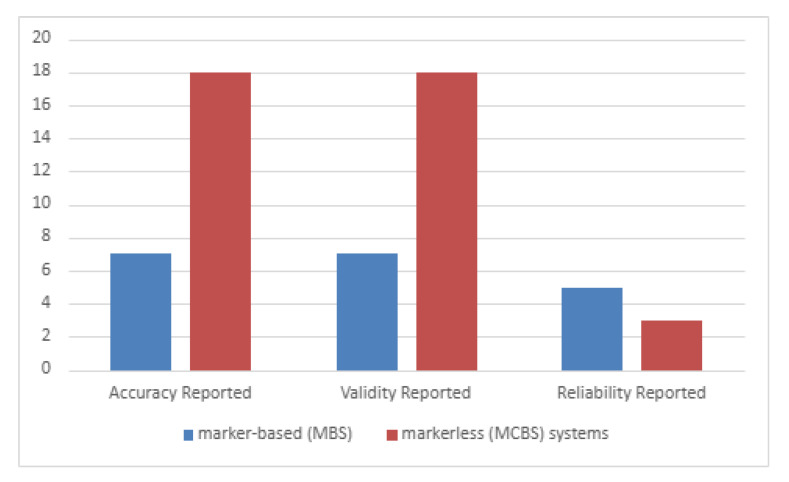
Accuracy, validity, and reliability of MBSs and MCBSs in ergonomic assessments.

Additionally, in the section below, we provide visual representations of the findings discussed (Table 11, Figure 2)

It is important to note that the counts in Table 10 reflect studies reporting quantitative metrics eligible for EBRO grading; the counts in Table 11 reflect studies that reported accuracy, validity, and reliability in any form (including qualitative descriptions).

Moreover, Figure 2 compares the reporting of accuracy, validity, and reliability for MBSs and MCBSs. MBSs show constantly higher reporting rates for accuracy and reliability, while MCBSs exhibit a lower reporting rate, particularly for reliability. However, in the studies of Eldar et al. [15] and Otto et al. [12], accuracy was not reported.

## 4. Discussion

This systematic review aimed to compare MBSs and MCBSs for assessing ergonomic risk analysis in terms of their accuracy, validity, and reliability in identifying ergonomic risks in workplace settings. Twenty quantitative studies [12,13,14,15,17,29,30,31,32,33,34,35,36,37,38,39,40,41,42,43] were included, with technologies ranging from high-fidelity optical systems to accessible AI-driven, vision-based platforms. The results demonstrate that while MBSs remain the reference gold standard for measurement accuracy, many MCBSs now approach acceptable thresholds for ergonomic evaluation and may offer practical benefits in real-world occupational contexts. These results are consistent with the findings in the previous literature [9,44].

Accuracy was the most consistently evaluated parameter, reported in 18 [13,14,16,17,29,30,31,32,33,34,36,37,38,39,40,41,42,43] of the 20 included studies. MBSs such as Vicon Bonita and BTS SMART-D consistently achieved joint-angle errors below 3°, with spatial precision in the sub-centimeter range. For example, Mehrizi et al. [16] reported joint-angle deviations of less than 3° when comparing a lifting task analyzed with both MBSs and MCBSs. In 2024, Jiang et al. [32] found that their deep learning model could estimate L5-S1 lumbar loads with RMSE values comparable to biomechanical modeling based on biplanar radiography. MCBSs showed more variability, but several achieved promising results. Moreover, Plantard [37] reported correlation coefficients between Kinect and Vicon joint angles ranging from r = 0.65 to 0.99 and normalized RMSE values for joint torque estimation ranging from 10.6% to 29.8%. Additionally, Liu et al. [33] recorded that 89% of detected joints fell within 20° of their reference markers, with landmark errors ranging from 6 to 12 cm (stereoscopic) and from 6 to 9 cm (ToF). In 2025, Bonakdar et al. [42] demonstrated strong correlations (r ≈ 0.95) and low RMSE values (6.5–9.9°) for hip, knee, and elbow joint angles and a normalized RMSE of ~9% for L5-S1 joint reaction forces compared to marker-based and inertial systems, while achieving 87% agreement in REBA scores. In the same year, Ojelade et al. [43] reported high classification accuracy for manual material handling tasks (≈93%) and hand configurations (≈96–97%), with moderate accuracy for lift origin classification (≈80–84%).

Furthermore, validity was assessed in 19 studies (out of which only 17 reported quantitively) [13,14,15,16,17,29,30,31,32,33,34,36,37,38,39,40,41,42,43], typically by comparing motion capture outputs with ergonomic risk scores derived from RULA, REBA, EAWS, or expert observers. However, some studies had moderate levels of evidence [12,14,15,36,37,38,41]. In another study, in 2016, Plantard et al. [38] found that RULA scores computed using Kinect data achieved 73–74% agreement with expert assessments in an industrial setting, with κ values ranging from 0.46 to 0.66. Additionally, Bortolini in 2018 [13] demonstrated that EAWS scores could be generated semi-automatically with posture detection using depth cameras, showing strong alignment with expert-coded scoring for postural sections B and C. Similarly, Manghisi et al. [34] developed a real-time AI-based model that produced REBA and EAWS scores with acceptable alignment to expert scorings. Alongside these, Bonakdar et al. [42] reinforced the validity of MCBSs by demonstrating agreement between their markerless system and gold-standard REBA outputs, while Ojelade et al. [43] supported validity through consistent performance across multiple RNN architectures and feature sets. In contrast, some studies, such as Wong et al.’s (2013) [41], relied more on visual comparison than structured validation, limiting confidence in their results.

Lastly, reliability was the least frequently reported measurement property. Only eight studies [16,29,31,32,34,37,42,43] evaluated it, out of which six included statistical evaluations. Plantard et al. (2017) [37] reported intra-class correlation coefficients (ICCs) ranging from 0.70 to 0.91 for joint angles captured using Kinect under different occlusion conditions. While Mehrizi [35] observed consistent outputs across multiple lifting trials, they did not provide formal reliability metrics. Many studies referenced repeatability or internal consistency without quantifying it [12,14,15,17,38,41]. However, Bonakdar et al. [42] reported high consistency of measurements across repeated trials, while Ojelade et al. demonstrated stable results across cross-validation folds with low variability between runs. Therefore, this lack of reliability testing in the majority of articles, particularly for MCBSs, remains a significant gap regarding their use in longitudinal or compliance-sensitive contexts.

In comparing these systems with traditional ergonomic tools, seven studies [14,15,34,36,37,40,42] evaluated how motion capture-derived outputs aligned with RULA, REBA, EAWS, or NIOSH scores. Most studies found that MCBSs could replicate expert-rated scores in static or semi-structured tasks. For example, Van Crombrugge et al. (2022) [40] achieved R^2^ values of 0.43 to 0.89 for key postural angles when comparing vision-based estimates with marker-based data. Similarly, Patrizi [36] showed that Kinect-based trunk and limb scores closely matched BTS-based motion capture when applying the NIOSH lifting equation. Traditional methods, while quicker and simpler to administer, were repeatedly shown to suffer from inter-observer variability. On the other hand, motion capture systems, particularly markerless ones enhanced with AI and depth sensing, offered continuous, objective risk detection in environments where observational tools may fall short.

Despite these advances, the evidence has important limitations. Reliability remains significantly underreported. Of the 20 included studies, only three [16,31,37] provided ICCs or similar repeatability metrics, and the majority did not address test–retest consistency at all. Moreover, several studies [12,13,14,17,31,36,40,42,43] used small sample sizes, sometimes fewer than 10 participants, which limits the generalizability of their findings. There was also considerable heterogeneity in study design, sensor placement, scoring systems, and outcome metrics, making it impossible to conduct a formal meta-analysis. As a result, pooled effect estimates and heterogeneity testing could not be performed.

From a methodological standpoint, this review was comprehensive but not without its own limitations. Only studies published in English and available as full texts were included, potentially excluding relevant English research papers. Although the COSMIN tool [27] provided a systematic way to evaluate measurement quality, it was applied across diverse study designs, and in some cases outcome interpretations required inference due to incomplete reporting.

These findings have meaningful implications for workplace ergonomics research. MCBSs, especially those using AI for pose estimation, can be considered suitable for ergonomic screening in structured or moderately complex industrial scenarios.

Their scalability, affordability, and automation potential make them attractive for organizations aiming to adopt continuous worker monitoring. However, accuracy, validity, and reliability must be considered for WMSD risk assessment in the workplace, and this was the objective of this systematic review.

## 5. Conclusions

This systematic review illustrates that not only are MCBSs advancing technologically, but they are also gaining traction as usable tools for ergonomic risk assessments in work settings. Compared to “observational” assessment tools such as RULA and REBA, MCBSs are more objective and allow for assessments in real time. They have several benefits in terms of accessibility, cost, and scalability, especially in real-world settings where MBSs may not be feasible due to the complexity of their set-up or subject movement. Though MBSs demonstrate more precision in their measurements (often with sub-centimeter and 3° joint-angle errors), their reliance on a controlled lab environment and specialized equipment hinders their generalizability to other contexts.

Markerless technologies that utilize depth-sensor-based and AI-based pose estimation and multi-view markerless reconstruction exhibit promising accuracies of approximately 2.31° ± 4.00° in joint-angle deviation while also demonstrating strong validity in their prediction of ergonomic scores. Most importantly, MCBSs had over 70% agreement with REBA and RULA codes, and reached a κ value of 0.71, suggesting that MCBSs are an effective tool for measurement and quantification of risk factors for ergonomic intervention.

Nonetheless, the review also identifies some important limitations, specifically regarding reliability. Only a few studies reported standardized reliability testing using intraclass correlation coefficients (ICCs), with ICC values ranging from moderate to excellent. The lack of formal reliable assessment results is concerning regarding the use of MCBSs in longitudinal tracking, compliance monitoring, and high-risk jobs, as repeatability is essential in these domains.

## Figures and Tables

**Table 1:** Search strategy in PubMed.

Database: PubMed	Search Strategy
P	(“workplace” OR worker * OR employee * OR industry OR manufacturing OR labor)
I	(“motion capture” OR “markerless” OR “marker-based” OR “motion analysis” OR kinematics OR biomechanics OR “3D analysis” OR “human movement” OR “body movement”)
C	(“assessment” OR “risk assessment” OR “task analysis” OR “manual handling” OR RULA OR REBA OR OWAS OR “postural analysis” OR “ergonomic tools”)
O	(“accuracy” OR “validity” OR “reliability” OR “evaluation” OR “comparison” OR reproducibility OR “measurement properties” OR “Reproducibility of Results” [MeSH])
Filters: Full text, English, humans, adults: 19+ years, from 1 January 2005 to 31 May 2025	

P: Population, I: Intervention, C: Comparison, O: Outcome, *: truncation (or wildcard) operator.

**Table 2:** Search strategy in WoS.

Database: PubMed	Search Strategy
P	TS = (“workplace” OR worker * OR employee * OR industry OR manufacturing OR labor OR job)
I	TS = ((“motion capture” OR “markerless” OR “marker-based” OR “motion analysis” OR kinematics OR biomechanics OR “3D analysis” OR “human movement” OR “body movement”))
C	TS = (“assessment” OR “risk assessment” OR “task analysis” OR “manual handling” OR RULA OR REBA OR OWAS OR “postural analysis” OR “ergonomic tools”)
O	TS = (“accuracy” OR “validity” OR “reliability” OR “comparison” OR reproducibility OR “measurement properties”)

P: Population, I: Intervention, C: Comparison, O: Outcome, *: truncation (or wildcard) operator.

**Table 3:** Search strategy in Science Direct.

Database: PubMed	Search Strategy
P	(Industry OR Work)
I	(Markerless OR Markerbase)
C	(Ergonomic OR Biomech)
O	(“Accuracy” OR “Reliability” OR “Validity”)
Filters: Date Range: 2005–2025	

P: Population, I: Intervention, C: Comparison, O: Outcome.

**Table 4:** Search strategy in IEEE EXplore.

Database: PubMed	Search Strategy
P	“Ergonomic” OR “Human Factors” OR “Biomechanical Risk” OR “Workplace Ergonomics” OR “Occupational Health” OR “Postural Risk” OR “Work-Related Musculoskeletal Disorders” OR “Ergonomic Assessment” OR “Physical Workload” OR “Occupational Biomechanics” OR “Postural Analysis” OR “Task Analysis”
I	“Marker-Based” OR “Markerless” OR “Optoelectronic System” OR “Optical Motion Capture” OR “Inertial Motion Capture” OR “Wearable Sensors” OR “Stereophotogrammetry” OR “Motion Tracking” OR “Kinematic Analysis” OR “Human Motion Analysis” OR “Computer Vision” OR “Pose Estimation” OR “3D Motion Analysis”
C	“Industry” OR “Work” OR “Occupational Setting” OR “Manufacturing” OR “Manual Labor” OR “Digital Human Model” OR “Workplace Safety” OR “Office Ergonomics” OR “Industrial Ergonomics” OR “Manual Handling” OR “Workplace Risk Assessment”
O	“Accuracy” OR “Validity” OR “Reliability” OR “Evaluation” OR “Performance Analysis”

P: Population, I: Intervention, C: Comparison, O: Outcome.

**Table 5:** Search strategy in PEDRo.

Database: PubMed	Search Strategy
Abstract and Title	“motion capture” markerless “marker-based” “motion analysis” biomechanics kinematics “3D analysis” “human movement” “body movement” workplace worker employee industry manufacturing labor job assessment “risk assessment” “task analysis” “manual handling” RULA REBA OWAS “postural analysis” “ergonomic tools” accuracy validity reliability evaluation comparison reproducibility “measurement properties”
Subdiscipline	Not Specified
Topic	Not Specified
Method	Clinical Trials
Published Since	1 January 2005
Language	English Only

**Table 6:** Eligibility criteria based on the PICO framework.

Category	Inclusion Criteria	Exclusion Criteria
Population	Studies involving working-age adults (18–65 years) performing occupational tasks in workplace settings, including industrial, healthcare, and service sectors.	Studies on non-occupational populations (e.g., athletes, children, and elderly individuals outside work contexts). Studies in recreational or non-workplace settings.
Intervention	Use of marker or markerless camera-based 3D motion capture for ergonomic risk assessment.	Studies utilizing 3D motion capture for non-ergonomic purposes (e.g., general biomechanics and sports performance).
Comparison	Not obligatory for inclusion; however, studies incorporating any form of comparison (e.g., 3D motion capture vs. traditional ergonomic assessments such as REBA and RULA) were considered.	
Outcomes	Accuracy, validity, and reliability of 3D motion capture in ergonomic risk evaluation.	Studies not reporting accuracy, validity, or reliability in relation to ergonomic risk assessment.
Study Design	Quantitative studies, including observational studies, validation studies, randomized controlled trials, cohort studies, and cross-sectional studies.	Qualitative studies, case reports, systematic reviews, or meta-analyses.
Language	Only studies published in English.	Studies published in languages other than English.

**Table 7:** Table of evidence.

Study (Author, Year)	Study Design	Setting	Technology Used	Comparison Method	Outcome Measures	Key Findings	Population
Abobakr et al., 2019 [29]	Validation study	Automotive manufacturing industry (field-based, ergonomic tasks)	Novel MCBS RGB-D sensor-based ergonomic risk assessment (CNN deep learning from depth images)	Kinetic skeleton datasets (built a synthetic data generation pipeline)	Joint-angle accuracy; RULA grand-score accuracy	Joint-angle error ±3.19° (±1.57°); RULA prediction accuracy 89% (κ = 0.71) indicating substantial agreement	Not explicitly reported
Boldo et al., 2024 [30]	Validation study	Industrial manufacturing environment (multi-camera human–machine interaction)	MCBS (distributed multi-camera 3D human pose estimation at the edge deployed (CNN-based)	MBS (infrared optical motion capture system, multi-camera setup)	Pose estimation accuracy; robustness to occlusion and multi-person tracking	High accuracy and robustness compared to MBSs; effective management of occlusion issues through multi-camera redundancy	Not explicitly reported
Bonakdar et al., 2025 [42]	Experimental validation	Lab experiment (University of Alberta Lab)	Markerless OMC (PoseChecker, RGB camera) + OpenSim	Marker-based OMC (Vicon), IMUs (Xsens), force plates, expert REBA	Joint-angle/JRF accuracy, REBA agreement	Back-angle r ≈ 0.95 vs. MBS-OMC/IMU; L5-S1 JRF r ≈ 0.91; REBA 87% match to MBS-OMC	8 healthy adults (4F/4M, ~25y)
Bortolini et al., 2018 [13]	Quasi-experimental study	Industrial workplace	MCBS (Multi-Kinect V2 depth camera network)	EAWS (observational)	EAWS risk index, task postures	MAS system using MCBS MoCap auto-computed EAWS sections accurately; time-saving and reliable for ergonomic evaluation	Automotive assembly operators
Brunner et al., 2022 [17]	Validation study	Lab (static postures)	MCBS (Kinect V2)	MBS (Vicon Bonita)	Joint-angle accuracy	Axial trunk rotation error ~14°; Kinect V2 showed issues with occlusions; risk of underestimation in posture evaluation	Human-sized mannequin
Eldar and Fisher-Gewirtzman, 2020 [15]	Experimental study	Simulated “third workplace”	MBS (VICON)	RULA + subjective feedback	Comfort, RULA, kinematic posture	Posture affected by workstation setup; MBS MoCap useful in design of ergonomic settings	3 seated e-workers
Fan et al., 2024 [31]	Validation study	Construction industry (field-based, construction tasks)	MCBS CV-based 3D human pose estimation (ConstructionPose3D dataset)	Non-CV-based 3D human pose estimation (solely on MuCo-3DHP)	Accuracy of pose estimation; REBA and RULA ergonomic scores	35% improvement in accuracy using construction-specific dataset compared to generic datasets; enhanced ergonomic assessment accuracy	7 construction workers (5 males, 2 females)
Jiang et al., 2024 [32]	Validation study	Lab (simulated lifting task)	MCBS (deep learning multi-cam)	MBS (Vicon) + Force Plate	L5-S1 load estimation (kinetics)	MCBS system estimated lumbar loads with high accuracy; force diff. ~14 N, moment diff. ~9 Nm	12 adults (avg. age: 24.2 years)
Li et al., 2018 [14]	Validation study	Lab (construction lifting task)	Marker-based 3D visualization (MBS)	Traditional observation (joint-angle comparison, risk rating comparison for body segments) + REBA/RULA	Accuracy, validity, risk ratings	3D visualization was comparable to motion capture and better than observation; useful for workstation redesign	3 healthy adults
Liu et al., 2022 [33]	Validation study	Lab (postural tasks)	MCBS (OpenPose + RGB-D)	MBS (OptiTrack Prime 13 motion capture system)	Accuracy (3D landmarks)	Average tracking error (cm): stereoscopic 6.96–12.47; ToF 5.57–9.31cm; sufficient for postural ergonomic assessment; OpenPose with RGB-D offers low-cost 3D solution	30 healthy adults (avg. age: 23.4 years)
Manghisi et al., 2017 [34]	Validation study	General industrial scenarios (real-time posture monitoring)	MCBS (Kinect V2)	Traditional visual assessment; expert rater assessment	RULA grand-score accuracy	Agreement proportion vs. MoCap = 0.97 (κ = 0.87), expert rater = 0.96 (κ = 0.84), indicating near-perfect agreement	Not explicitly reported
Mehrizi et al., 2018 [16]	Validation study	Lab (symmetrical lifting tasks)	MCBS (computer vision)	MBS (Optical; Motion Analysis Corp., Santa Rosa, CA; 100 Hz, 45-marker setup)	Accuracy (joint angles), validity	Joint angles differed <3° from MBS; accurate enough for ergonomic risk estimation in lifting scenarios	12 healthy males (47.5 ± 11.3 years)
Ojelade et al., 2025 [43]	Experimental validation + ML classification	Lab experiment (Virginia Tech lab)	Azure Kinect markerless MoCap + RNNs (Bi-LSTM, GRU, BGRU)	RNN models and feature sets compared	Accuracy, precision, recall, and F1 for task/hand/lift classification	Best ≈ 93% accuracy; TOP-80 > TOP-60; hand ≈ 97% acc., lift origin ≈ 80–84%; GRU+TOP-80 efficient	36 healthy adults (14 F), right-handed
Otto et al., 2019 [12]	Evaluation study	Lab (automotive setting)	MCBS (Kinect V2)	Expert evaluation (EAWS)	Validity, feasibility	9 out of 11 EAWS postures accurately captured; sitting and standing performed well; lying poses not reliably tracked	3 adult volunteers
Patrizi et al., 2016 [36]	Validation study	Real working tasks	MCBS (Kinect V1)	MBS (BTS)	Posture score agreement	MCBS Kinect system provided scores close to BTS system; supports Kinect as cost-effective alternative	3 adult participants
Plantard et al., 2016 [38]	Observational study	Lab + real workplace	MCBS (Kinect V2)	Human experts + Vicon (MBS)	Validity (RULA), accuracy	Kinect data accurately predicted RULA scores; robust in cluttered industrial settings	12 volunteers + 7 workers
Plantard et al., 2017 [37]	Validation study	Simulated lab work tasks	MCBS (Kinect V2)	MBS (Vicon)	Accuracy, reliability, validity	Kinect showed high correlation with MBS; feasible for ergonomic risk assessment in occluded environments	12 adults (30.1 ± 7.0 years)
Seo et al., 2019 [39]	Comparative study	Construction tasks	MCBS (RGB-D, Stereo, Multi-cam)	MBS (Optotrak)	Joint-angle error, feasibility	Vision-based systems had 5–10° error; angular sensor ~3°; suitable for field posture screening with minor trade-offs	
Van Crombrugge et al., 2022 [40]	Validation study	Assembly lab simulation	MCBS (RealSense + Detectron2)	MBS (VICON)	Joint angles, REBA	3D skeletons from triangulated 2D joint detection estimated key joint angles with moderate error	1 adult male
Wong et al., 2013 [41]	Validation study	Operating theatre simulation	Hybrid (MCBS vision and Biomotion + Wearable (IMU)	MBS (BTS SMART-D)	Accuracy (pose estimation), tracking fidelity	Combining visual head tracking and inertial data achieved accurate motion capture under occlusion	Surgical team (simulated)

MCBS = Markerless Camera-Based System, MBS = Marker-Based System, RGB-D = Red, Green, Blue + Depth Camera, OMC = Optical Motion Capture, IMU = Inertial Measurement Unit, CV = Computer Vision, EAWS = European Assembly Worksheet, RULA = Rapid Upper Limb Assessment, REBA = Rapid Entire Body Assessment, MAS = Motion Analysis System, OpenPose = OpenPose Algorithm (open-source real-time multi-person keypoint detection library), Detectron2 = Detectron2 Algorithm (computer vision framework for object detection and keypoint estimation), MuCo-3DHP = Multi-Person Composites of 3D Human Pose Dataset, ConstructionPose3D = Construction-Specific 3D Human Pose Estimation Dataset, OptiTrack = Brand of Motion Capture Systems by NaturalPoint Inc. (e.g., Prime 13 Model), Optotrak = Optical Motion Tracking System by Northern Digital Inc. (NDI), Vicon = Optical Motion Capture System (various models: MX, MX13, Bonita, etc.), BTS SMART-D = Optical Motion Analysis System by BTS Bioengineering.

**Table 8:** Summary of accuracy, validity, and reliability in included studies.

Study	Accuracy (Metrics)	Validity (Comparison Basis)	Reliability	Parameters Assessed
Abobakr et al. (2019) [29]	High precision from AI-based system; RMSE values within clinical acceptability	Compared estimated joint trajectories against labelled datasets	Not evaluated	Whole-body joint angles, AI vision system performance
Boldo et al. (2024) [30]	High agreement reported; 3D postural estimates aligned with ergonomic models	Compared visual system output with expert ergonomic review	Reliability discussed qualitatively; no formal metrics	Multi-joint angles, postural scoring via AI
Bonakdar et al. (2025) [42]	Back-angle r ≈ 0.95 vs. MB-OMC/IMU (RMSE 6.5–9.9°); L5-S1 JRF r ≈ 0.91 (nRMSE ~9%); REBA 87% match to MB-OMC	Compared with marker-based OMC, IMUs, force plates, and expert visual REBA	High consistency across trials; statistical agreement with MB-OMC	Joint angles, joint reaction forces (L5-S1, hip, knee, elbow), REBA scores
Bortolini et al. (2018) [13]	Postural scores from MAS matched expert EAWS sections; semi-automated scoring evaluated qualitatively	Postural scoring compared with manually scored EAWS	Reliability discussed, not statistically assessed	Assembly line posture, EAWS components
Brunner et al. (2022) [17]	Mean deviation: 14.04° in axial rotation, maximum deviations in self-occluded positions	Compared MCBS Kinect v2 against MBS Vicon Bonita	Not evaluated	Static upper body poses; trunk rotation accuracy
Eldar (2020) [15]	Posture and comfort compared across work configurations; scoring deviations not numerically detailed	RULA scoring and subjective feedback used to assess workstation ergonomics	Not evaluated	Neck, spine, and shoulder postures during tablet use
Fan et al. (2024) [31]	Large dataset used; model accuracy tested with human pose database; joint detection error metrics reported	High model alignment with reference poses in construction simulation	Reliability discussed qualitatively; no formal repeatability testing	Joint center detection, construction task simulation
Jiang et al. (2024) [32]	RMSE between estimated and reference L5-S1 loads calculated using biplanar radiography and biomechanical modeling	Good agreement between estimated and reference spinal loads; qualitative and quantitative validation	Not formally tested; mentions consistency over trials	L5-S1 spinal load (force and moment components)
Li et al. (2018) [14]	*Numeric accuracy reported.* Overall corr. for vertical angles r = 0.80 (up to 0.94 for hand/lower arm/upper and lower leg); trunk flexion (S3) avg. diff. = 10.24°, avg. error = 11%; 14/20 angles < ±14% error	Workstation assessment validity supported by ergonomic expert consensus, no statistical evaluation	Not evaluated	41 joint angles; lifting task; explicit difference/error equations reported for comparisons
Liu et al. (2022) [33]	3D landmark error: typically, ~3–12 cm depending on landmark and posture; largest errors at hips in occluded postures (e.g., ~20–35 cm for mid-hip/hips in some sitting/stoop conditions); use when you want the per-landmark nuance, otherwise keep the averages above	Compared landmark detection using OpenPose vs. marker-based motion tracking and inertial sensors	Not evaluated	Whole-body 3D body landmarks during static postures
Manghisi et al. (2017) [34]	Acceptable deviation from reference; REBA/EAWS estimates generated in near-real time	AI-driven model tested against expert EAWS scorings	No formal metrics, but repeatability discussed	Joint angles, ergonomic scoring (REBA, EAWS)
Mehrizi et al. (2018) [16]	Joint-angle error <3° compared to gold standard; strong agreement in sagittal plane	Angle accuracy validated against optical MBS	Qualitative repeatability noted; no formal metrics	Symmetrical lifting task: joint angles, body segment tracking
Ojelade et al., 2025 [43]	Task classification = 93% agreement; hand classification ≈ 96–97%; lift origin ≈ 80–84% (F1-scores similar)	Compared performance across RNN models (Bi-LSTM, GRU, BGRU) and feature sets (TOP-60, TOP-80)	Stable across cross-validation folds; low variance between runs	Task type classification, hand configuration classification, lift origin classification
Otto et al. (2019) [12]	Not reported numerically; visual posture accuracy confirmed for 9/11 EAWS movements	Ergonomic classification visually compared with EAWS expert assessments	Not tested; performance inconsistencies noted in crouched/lying positions	Postural assessment (EAWS), standing/sitting/crouching
Patrizi et al. (2016) [36]	Joint-angle deviations from reference system (BTS); correlation reported between Kinect and BTS scores	NIOSH lifting metrics and RULA compared; strong qualitative match	Qualitative repeatability noted; no formal metrics	Trunk and arm angles, NIOSH risk multipliers
Plantard et al. (2016) [38]	RMSE in RULA score: 0.22–0.68; correlation of shoulder angles: r = 0.68–0.98 across conditions	Compared RULA scores computed from Kinect with expert assessments; κ = 0.46–0.66; 73–74% agreement in real work	Not formally assessed; limitations noted in occluded conditions	RULA scores, shoulder and elbow joint angles
Plantard et al. (2017) [37]	Joint-angle cross-correlation with Vicon: r = 0.65–0.99; RMSE for joint torque values (nRMSE: 10.6–29.8%)	Joint torques and angles compared with Vicon; acceptable RMSE and correlation; residual force validation under 3.5%	Mean Kinect joint reliability: 0.70–0.91, depending on occlusion level	Shoulder/elbow joint angles, joint torques, residual forces
Seo et al. (2019) [39]	Joint-angle error: 5–10° for vision systems, 3° for encoder-based systems	Accuracy validated against MBS (Optotrak) data	Not evaluated	Construction task joint tracking (shoulder, trunk)
Van Crombrugge et al. (2022) [40]	RMS joint-angle error: 12° using combined triangulation; R^2^ values: 0.43–0.89 for key REBA angles	Joint-angle comparison with VICON system; REBA angle alignment visualized	Not evaluated	Shoulder, elbow, and trunk joint angles relevant to REBA
Wong et al. (2013) [41]	*Numeric accuracy reported (pixels) for head detection*, not joint-angle RMSE: mean (±SD) error range ≈ 7.9–85.3 px across single-/multi-person conditions	Compared head pose tracking with BTS system in simulated surgery	Not evaluated	Head tracking under occlusion; IMU + visual detection

RMSE = Root Mean Square Error, nRMSE = Normalized Root Mean Square Error, ICC = Intraclass Correlation Coefficient, κ = Cohen’s Kappa Coefficient (measure of inter-rater agreement), r = Pearson’s Correlation Coefficient, R^2^ = Coefficient of Determination, L5-S1 = Lumbosacral Joint Between the Fifth Lumbar and First Sacral Vertebra, RNN: Recurrent Neural Network, Bi-LSTM: Bidirectional Long Short-Term Memory, GRU: Gated Recurrent Unit, BGRU: Bidirectional Gated Recurrent Unit, F1: F1-Score, ML: Machine Learning, OpenSim: Open-Source Biomechanical Modeling and Simulation Software, Xsens: Brand of inertial measurement unit motion capture systems.

**Table 9:** Summary of risk of bias assessment based on COSMIN tool (I = Inadequate, D = Doubtful, A = Adequate, V = Very Good). (-) indicates that the property was not assessed in the study.

Study	Reliability	Validity	Accuracy	Conclusion	Rating
Abobakr et al. (2019) [29]	-	V	V	AI-based assessment with strong accuracy validation	V
Boldo et al. (2024) [30]	A	V	A	High validity and accuracy; significant reliability concerns	V
Bonakdar et al., (2025) [42]	A	A	A	High consensus of accuracy, validity, and reliability	A
Bortolini et al. (2018) [13]	A	V	A	Strong validity but lacks formal test–retest reliability	A
Brunner et al. (2022) [17]	D	A	A	High validity but significant trunk rotation errors	D
Eldar (2020) [15]	D	V	I	Some reliability issues; accuracy not reported	A
Fan et al. (2024) [31]	A	V	A	Large dataset validation but lacks test–retest reliability	A
Jiang et al. (2024) [32]	A	V	V	Strong validity and accuracy; minor reliability concerns	V
Li et al. (2018) [14]	D	D	A	Lacked reliability testing but accuracy is adequate	A
Liu et al. (2022) [33]	-	V	A	High validity, accuracy is acceptable	A
Manghisi et al. (2017) [34]	A	V	A	Strong validity with minor inter-rater concerns	A
Mehrizi et al. (2018) [16]	A	V	V	High validity and accuracy, minimal bias risk	V
Ojelade et al., 2025 [43]	A	V	V	High accuracy and reliability, moderate validity	V
Otto et al. (2019) [12]	I	A	I	Limited reliability; validity is acceptable	D
Patrizi et al. (2016) [36]	-	A	A	Minor validity concerns, overall acceptable	A
Plantard et al. (2016) [38]	D	A	A	Adequate validity but lacks robust reliability testing	A
Plantard et al. (2017) [37]	A	A	V	Some reliability concerns but strong accuracy	A
Seo et al. (2019) [39]	-	V	A	Strong validity, self-occlusion challenges exist	A
Van Crombrugge et al. (2022) [40]	-	V	A	High validity but lacks dynamic testing	A
Wong et al. (2013) [41]	D	A	A	Adequate validity; lacks strong reliability testing	A

**Table 10:** EBRO levels of evidence by outcomes.

Measurement Property	No. of Studies	EBRO Level of Evidence	Summary Justification	Certainty Level
Accuracy	18	A2	Consistent findings from multiple high-quality cohort/validation studies with RMSE < 5°, high correlations (r > 0.85), and joint-angle errors under 10° in most conditions	High
Validity	17	B	Moderate to strong agreement with expert-rated RULA/REBA/EAWS scores; κ up to 0.71; studies well-aligned with ergonomic constructs	Moderate
Reliability	6	C	Limited statistical evaluation (ICCs in a few studies); other studies described repeatability qualitatively or not at all; inconsistent or missing reporting	Low

**Table 11:** Reporting of measurement properties across studies.

Study	Accuracy Reported	Validity Reported	Reliability Reported
Abobakr et al., 2019 [29]	1	1	0
Boldo et al., 2024 [30]	1	1	1
Bonakdar et al., 2025 [42]	1	1	1
Bortolini et al., 2018 [13]	1	1	0
Brunner et al., 2022 [17]	1	1	0
Eldar and Fisher-Gewirtzman, 2020 [15]	0	1	0
Fan et al., 2024 [31]	1	1	1
Jiang et al., 2024 [32]	1	1	0
Li et al., 2018 [14]	1	0	0
Liu et al., 2022 [33]	1	1	0
Manghisi et al., 2017 [34]	1	1	1
Mehrizi et al., 2018 [16]	1	1	1
Ojelade et al., 2025 [43]	1	1	1
Otto et al., 2019 [12]	0	1	0
Patrizi et al., 2016 [36]	1	1	1
Plantard et al., 2016 [38]	1	1	0
Plantard et al., 2017 [37]	1	1	1
Seo et al., 2019 [39]	1	1	0
Van Crombrugge et al., 2022 [40]	1	1	0
Wong et al., 2013 [41]	1	1	0
Total	18	19	8

## Data Availability

All titles and abstracts resulting from the database search were directly imported into Rayyan for screening and management. The datasets generated and analyzed during the current study are available from the corresponding author upon reasonable request. Due to licensing restrictions from the database providers, raw data (i.e., full records retrieved from proprietary databases) cannot be publicly shared.

## Abbreviations

The following abbreviations are used in this manuscript:
